# The emerging application of ultrasound technology in pediatric bone fractures: Clinical application, related issues and development prospect

**DOI:** 10.1002/pdi3.69

**Published:** 2024-05-28

**Authors:** Xiang Li, Xing Liu, Mingyan Shi, Man Zhang, Peikang Wang, Xinkai Zhang

**Affiliations:** ^1^ Department of Orthopedic Ministry of Education Key Laboratory of Child Development and Disorders National Clinical Research Center for Child Health and Disorders China International Science and Technology Cooperation Base of Child Development and Critical Disorders Chongqing Key Laboratory of Pediatrics Children's Hospital of Chongqing Medical University Chongqing China

**Keywords:** callus, fracture healing, pediatric fracture, ultrasonography

## Abstract

Ultrasonography has begun to be applied to the examination of fractures in recent years owing to its safety, noninvasiveness, portability, and high sensitivity. The subcutaneous soft tissue of children is thinner than that of adults, children's bones have a high level of elasticity and a low level of brittleness, and the pediatric fractures heal quickly and produce more callus, therefore ultrasonography is a more suitable examination in pediatric bone fractures. In this paper, we intend to review the mechanism, performance, and examination effect of ultrasound examination in bone fractures, analyze the advantages and disadvantages between ultrasound imaging and X‐ray imaging, and further propose an outlook for the application of ultrasound technology in pediatric bone fractures.

## 1 INTRODUCTION

Ultrasound technology has evolved over more than a century to include A, B, M, D and three‐dimensional ultrasound, and has achieved significant breakthroughs in various fields, its performance in medical is more outstanding in now days. In recent years, more and more scholars have begun to study the application of ultrasonography in the examination of fractures. The incidence of fractures in children is approximately 3%,^1^ pediatric fractures are different from adult fractures. Characteristics of pediatric fractures include greenstick fracture, easy damage to the epiphyseal plate, subperiosteal fractures, and cleft fractures. Related studies have shown that some children may not have obvious pathological signs after the occurrence of fractures and may be misdiagnosed using conventional radiography.^2^, ^3^, ^4^, ^5^, ^6^ In addition, conventional radiography inevitably has radiation damage and is riskier for children.^7^, ^8^, ^9^ During fracture healing, several times of examination are usually required to check the healing tissue around the fracture end. Related studies have shown that children in growth and development are more sensitive to radiation than adults, and radiation exposure poses a correspondingly greater hazard to children.^10^, ^11^, ^12^, ^13^ X‐ray examination also cannot be observed dynamically in real time, and the bedside X‐ray machine often can only emit X‐rays from a single angle, which has certain limitations. Ultrasonography, on the other hand, provides better imaging of soft tissue injuries, it can be used to reduce the risk of missed diagnoses in pediatric incomplete fractures, which have poorly visible appearance. In addition to high sensitivity, ultrasonography also has the features of no radiation damage, good soft tissue and vascular imaging, the ability to perform ultrasound‐guided closed manipulative repositioning, and earlier observation of callus formation. Therefore, ultrasonography plays an increasingly critical role in the diagnosis and management of pediatric fractures.

## 2 OVERVIEW OF PEDIATRIC FRACTURES

The bone quality of children is different from that of adults: children have more immature bones but adults have more mature bones. Children's bones contain fewer inorganic salts than adults, and more organic substances such as collagen than adults, so it has the characteristics of being more elastic and less brittle. The presentation of fractures also varies considerably by maturity of bones. Because of the above‐mentioned characteristics of children's bones, when external forces are applied, children's bones are soft and easy to bend, thus dispersing the effect of stress to reduce the chance of damage. Because of this feature, children have a higher incidence of greenstick fracture, this is when a bone is bent and breaks along only one side, like a young stick of wood. Therefore, the pathological signs of fractures in some children may not be obvious.^14^ In addition, the epiphyseal plate, which is unique to children's bone growth, is composed of cartilage cells and is more resilient, and is an important tissue for bone growth and development in children. However, epiphyseal separation injury often occurs in pediatric fractures, which may cause serious complications such as bone growth arrest, asymmetric growth, and growth retardation. The periosteum of children's bones has a strong osteogenic ability, so when fractures do occur, they heal more quickly in children than in adults, and the children's callus production is more pronounced during the healing process. This set of characteristics makes ultrasonography more advantageous in the examination in pediatric fractures.

## 3 PRINCIPLE OF ULTRASONOGRAPHY IN FRACTURE EXAMINATION

Ultrasound examination of fractures is based on the difference in acoustic impedance between soft tissue, callus, fracture end hematoma and bone cortex, and ultrasound is reflected to form a distinct acoustic interface between them, which can be clearly identified on the sonogram.^15^, ^16^, ^17^, ^18^

During an ultrasound examination, a multifaceted sweep of the long axis add short axis is usually performed. On the ultrasound sonogram, the normal bone cortex appears as hyperechoic, well‐defined, high‐bright and smooth lines. To optimize the brightness and detail of the bone cortex in the sonogram, the angle of the ultrasound beam and the ultrasound transducer should be adjusted to be as perpendicular as possible to the surface of the bone. When bone fracture occurs, the continuity of the injured bone cortex is interrupted and the fractured end may become angulated. It can be specifically represented on the long‐axis diagram of the sonogram as a highlighted smooth straight lines interruption and may appear as two non‐parallel straight lines. In addition, abnormal hyperplasia and thickening of the periosteum around the fracture end can be observed on the sonogram, and scanning with Color Doppler ultrasound, it is possible to observe blood flow signals to clarify the blood supply. Meanwhile, wrapped hypoechoic images can usually be clearly observed around the fracture end, which is a sonographic manifestation caused by soft tissue injury and vascular damage around the fracture end.

## 4 ADVANTAGES OF ULTRASONOGRAPHY IN FRACTURE EXAMINATION

### 4.1 Sensitivity

Ultrasound examination provides a better representation of the bone cortex on the sonogram and can clarify whether a fracture has occurred. A study of children aged 4–11 years with forearm fractures who underwent ultrasound achieved a correct diagnosis rate of over 99%.^1^ Multiple measurements of the fracture in different axes and angles are possible with ultrasonography. In addition, the presence of vascular damage and soft tissue damage can be observed on the sonogram, which can help diagnose. In contrast, the most common traumatic injuries in emergency centers are foot and ankle injuries, and only about 15% of patients can be definitively detected using conventional radiography.^3^ A retrospective study examined 268 patients with negative X‐ray findings for foot and ankle injuries by the high‐resolution ultrasound and found 24 patients with occult fractures, which was attributed to the fact that ultrasound can provide important information about soft tissue injuries and localized regional cortical discontinuities to assist in confirming the diagnosis.^4^ Similarly for the sternum, ultrasonography can be applied equally effectively due to its superficial location under the skin. In one study, two children with blunt trauma to the chest had normal chest X‐rays, whereas ultrasonography showed a sternal fracture with distal dorsal displacement of the fracture in the first child and a transverse sternal fracture without displacement in the second child. Finally, magnetic resonance imaging confirmed sternal fractures in two children and excluded concomitant injuries to the soft tissue adjacent to the sternum.^2^ This confirms that ultrasound has a better sensitivity than X‐ray for the examination of specific areas. At the same time, ultrasound technology also has good prospects for development. Novel quantitative vibroacoustic methods have been used to distinguish fractured bone from intact bone by exploiting the vibrational properties of bones with different physical properties.^19^, ^20^ Some studies have also proposed the use of ultrasound capture techniques instead of conventional radiography for fracture examination. By using this measurement, they compared the detection between uncut bone, versus cut bone cut 2 mm, 5 and 10 mm deep, using a *t*‐test at the *α* = 5% level, and the conclusion suggested a significant difference.^21^

### 4.2 Ultrasound‐guided operation

A common fracture in children is the ulnar radius fracture, which accounts for approximately 13% of all pediatric fractures.^22^ A study of 80 forearm fractures managed by closed reduction with an average follow‐up of 5 years and 9 months found that 92% of them had good or excellent results, 8% had fair results, and no poor results.^23^ Treatment with external fixation by plaster after manual repositioning can be applied to most of the fractures with satisfactory results. In a study of 458 children with forearm fractures, 289 of whom underwent closed reduction without real‐time imaging assistance and 169 under ultrasound guidance, 5% of the former patients required continued surgical treatment and only 1% of the ultrasound group required surgical treatment.^24^ Another study examined 27 children with distal humeral epiphyseal fractures, all of whom underwent ultrasound‐guided closed reduction and all achieved good or excellent functional and cosmetic recovery after healing as assessed by Flynn's criteria assessment.^25^ In another study, 24 patients with acute metacarpal fractures were subjected to a randomized controlled trial, and all patients were divided into experimental and control groups of 12 patients each, with ultrasound‐guided closed reduction and fixation in the experimental group and C‐arm fluoroscopy‐assisted fixation in the control group. The efficacy of the two groups was tracked and it was found that there was no significant difference in the success rate of fracture closed reduction and the mean fracture healing time between the experimental group and the control group.^26^ It suggested that the superficiality of the ulnar radius site is more suitable for ultrasound examination.^24^, ^27^, ^28^, ^29^, ^30^, ^31^ The ultrasound‐guided repositioning allows real‐time monitoring during the repositioning process and timely adjustments, as well as repeated examinations without radiation hazards.

In recent years, ultrasonography has applied in surgical treatment of bone fractures. The ultrasound‐guided fixation has better results compared to traditional C‐arm fluoroscopic‐assisted fixation. In a study of 45 children who underwent the same procedure with X‐ray assistance and ultrasound assistance, we found that the use of ultrasound‐assisted surgery not only significantly reduced radiation exposure, but also showed no significant difference in postoperative recovery between the two based on the Constant–Murley shoulder score.^32^ In another recent study, 56 children with forearm fractures were treated with ultrasound guidance and fluoroscopy guidance, and the results showed statistical differences in operative time, fluoroscopy time, and radiation dose between the two groups, with the ultrasound group performing better and with no differences in elbow function between the two groups at final follow‐up.^33^ This suggests that ultrasound technology also plays an important role in the surgical treatment in pediatric fractures and holds promise as an alternative to conventional X‐ray to reduce radiation exposure to patients and health care workers during surgery.^34^, ^35^, ^36^, ^37^

In addition, ultrasound‐guided ulnar nerve block (UGUNB) has been successfully used to reset finger bone fractures, this study indicates that UGUNB is effective for pain control in the pediatric emergency setting and can effectively perform fracture repositioning of the phalanges.^38^ In another study, 45 children with displaced supracondylar fractures of the humerus were studied, and the fractures were repositioned under ultrasound guidance while the ulnar nerve was detected by ultrasound, and no complications such as ulnar nerve injury or elbow inversion were found in any of the children.^35^ In addition, several studies have demonstrated the promising use of ultrasound in pediatric fractures associated with nerve damage.^39^, ^40^, ^41^, ^42^

### 4.3 Monitoring fracture healing

**FIGURE 1:**
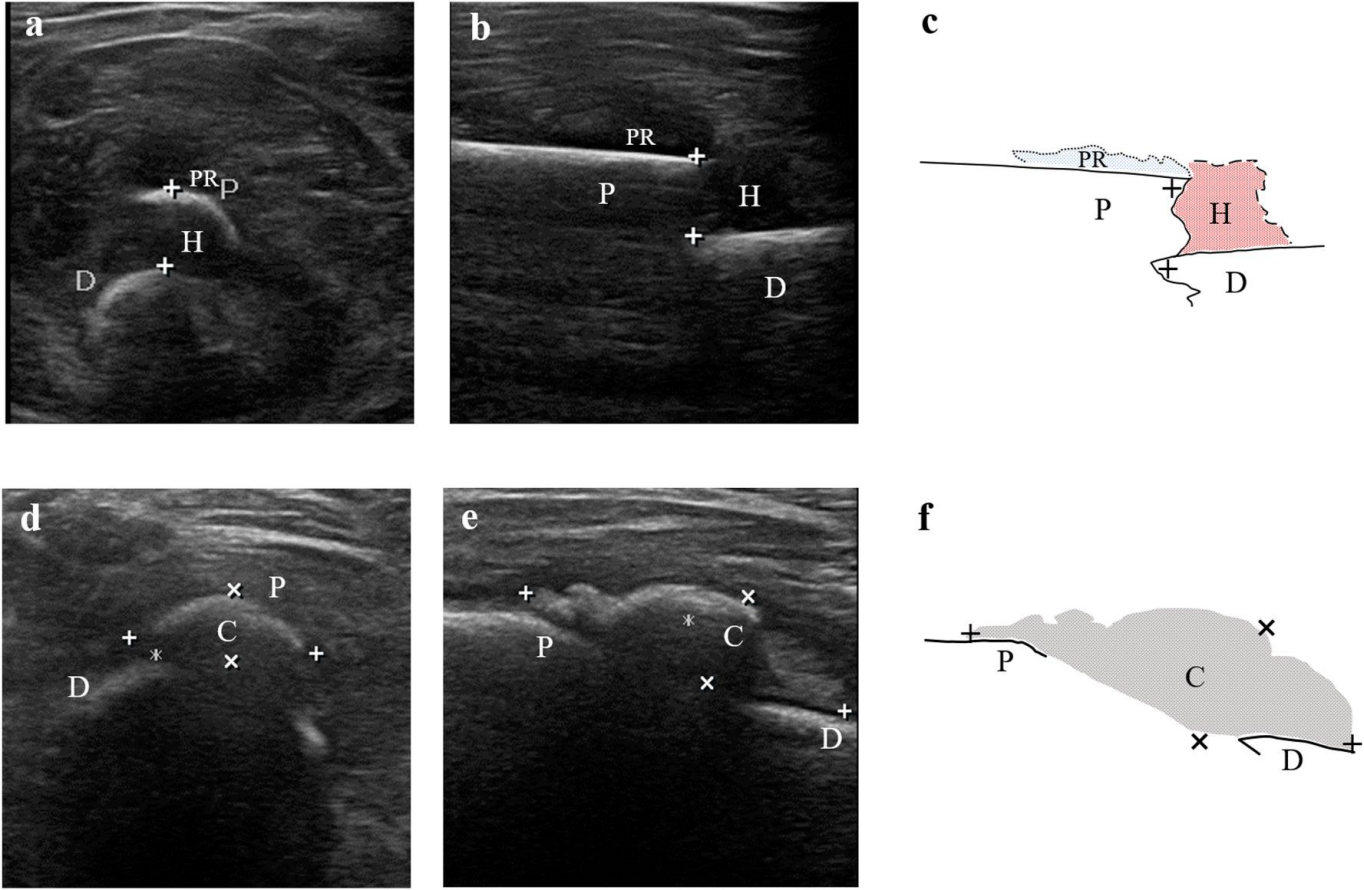
Left middle femur fracture of a 1‐year‐old boy within 1 day after injury (A–C) and after 1 week with conservative treatment (D–F). (A) Cross‐sectional ultrasound sonogram (B) Longitudinal ultrasound sonogram (C) Demonstrate the schematic diagram of fracture within 1 day after injury in the longitudinal section (D) Cross‐sectional ultrasound sonogram (E) Longitudinal ultrasound sonogram (F) Demonstrate the schematic diagram of fracture after 1 week with conservative treatment in the longitudinal section. C, callus; D, distal end; H, hematoma; P, proximal end; PR, periosteal reaction. The distance between fracture ends (plus sign in A–C). Callus length (plus sign in D–F). Callus thickness (multiplication sign).

Fracture healing is generally divided into three phases: hematoma mechanization phase, primary callus formation phase, and callus remodeling plasticity phase. It was also shown that the growth of callus around the fracture end could be observed more effectively on X‐ray only when the callus around the fracture end growed to a calcium salt content of 25% or more after the fracture occurs.^43^ In a study of 21 patients with tibial fractures examined under ultrasound and X‐ray, ultrasound was found to detect callus earlier than X‐ray.^44^ In our study, we found that callus production was observed using ultrasonography 1 week after the fracture occurred in children (Figure 1). This is due to the fact that ultrasound has good imaging of soft tissue and soft bone fragments compared to X‐ray examination. A prospective study of 50 children treated for long bone fractures of the arm, forearm, thigh and calf was performed by ultrasound, and the results obtained by ultrasound were compared with radiographic measurements and subjective assessment of healing tissue quality. The differences between the results obtained by ultrasound and X‐ray were not statistically significant. Thus, it was shown that ultrasound is highly efficacious in assessing healing tissue formation after long bone fractures in children and has the potential to replace radiography.^45^

## 5 DISADVANTAGES OF ULTRASONOGRAPHY IN FRACTURE EXAMINATION

### 5.1 Ultrasonographic artifacts

The basic principle of sonogram is the reflection of the ultrasound signal by different tissue. Therefore, during the examination, when ultrasound signals are blocked or interfered by foreign matter, artifacts will be shown on the sonogram.^46^ A common artifact in ultrasound examination of fractures is a pseudo‐fracture line, where the examined area appears as a fracture on the sonogram but is actually a sonogram of other tissue.^47^ We believe that artifacts that may arise during the examination include the posterior acoustic shadow produced by nutrient vessels, epiphyseal plates, eroded bone cortex, and seed bone, small bone, or other foci of calcification. It is worth mentioning that some of the postoperative changes, such as the installed fixators, bone tunnel suture anchors, and partial bone resection or remodeling may cause artifacts to appear on the sonogram when examined by ultrasound.

### 5.2 Poor examination performance at special areas

Some scholars had found that ultrasound was less effective in examining fractures in proximal joint areas.^11^ Meanwhile, several scholars had pointed out that, especially for structural injuries within the knee joint, the visualization of ultrasound examination is often poor and does not provide a strong imaging support basis for clinical diagnosis of fractures.^48^, ^49^, ^50^, ^51^ A joint is composed of joint surface, joint capsule and joint cavity, and the joint capsule is composed of tough connective tissue, which forms the joint cavity with articular cartilage, and there is also synovial fluid in the joint cavity. All of these may be the factors that make poor performance in ultrasound examination.

### 5.3 Ultrasonography is limited by multiple factors

Compared with X‐ray examination, we found that ultrasound examination has more strict limits, such as: thicker subcutaneous fat and soft tissue, indication of local skin breakdown or even infection, and the use of external or internal fixation, etc. Children with these factors are generally not suitable for ultrasound examination. In addition, we also noted that the accuracy of ultrasound examination is associated with the level of experience of the operator, which undoubtedly increases the technical requirements for the operator. During ultrasound examination, it is usually necessary for the operator to press the ultrasound probe to apply pressure in order to obtain better sonographic performance, and in children with fractures, such an operation can cause increased pain and increased discomfort during the examination, which may further affect the ultrasound imaging performance.

## 6 DISCUSSION

Fracture is a common injury in children. Children's bones have more organic material such as collagen and less inorganic material in them, therefore, the bones of children in growth and development have unique physiological characteristics, whether analyzed in terms of injury performance and degree of healing. It is also the hidden nature of some of the injuries and the rapid healing rate of fractures in children that has led many scholars to focus on the application of ultrasound technology in children with bone fracture. Children have thinner subcutaneous soft tissue than adults, so ultrasound may have better imaging results. At the same time, for children who are at a critical stage of bone growth and development, radiation‐free ultrasound is obviously more acceptable. In addition, the flexibility and ease of transport of portable ultrasound machines are clearly more advantageous than the X‐ray machines used for traditional examinations. Scholars generally agree that ultrasound can be better utilized in emergency medicine.^24^, ^31^, ^52^, ^53^, ^54^, ^55^, ^56^

It is also undeniable that ultrasonography also has some limitations. However, considering its good performance in children with fracture, most scholars now believe that there is some value in using ultrasound to examine common long bone fractures in children. For now, ultrasound imaging and X‐ray imaging have their own advantages and unavoidable disadvantages (Table 1), it is not a bad idea to use ultrasound examination as a complementary tool to X‐ray examination.

**TABLE 1:** The pros‐cons comparison between x‐ray imaging and ultrasound imaging.

	Advantages	Disadvantages
Ultrasound	High sensitivity	Ultrasonographic artifacts
	Ultrasound‐guided operation	
	Monitoring early healing	
	Safety	
	Soft tissue imaging	Inapplicable at special sites
	Portability	Harder operation
	Dynamic observation	
X‐ray	Quick	Radiation damage
	Widely applicable	Poor portability
	Easier operation	Poor soft tissue imaging

Ultrasound technology is still in a phase of rapid development, and the development of three‐dimensional ultrasound also offers new possibilities for the application of ultrasound technology in fractures.^57^, ^58^, ^59^ Not only the observation of bone, but also the excellent performance of ultrasonography in soft tissue imaging is the key to its uniqueness. The portability and affordability of ultrasound technology is destined to make it stand out among the many imaging examinations of fractures. The perfect fit of ultrasonography in pediatric fractures is bound to help more scholars focus on the excellent developmental prospects of this technology, and it is believed that the application of ultrasonography in fractures will be even more brilliant in the near future.

## AUTHOR CONTRIBUTIONS

Xiang Li and Xing Liu were involved in the conception, design of the project, and made the critical revisions. Xiang Li wrote the original paper and Xing Liu reviewed, revised it. Xiang Li participated in the ultrasound examination. Mingyan Shi, Peikang Wang, Xinkai Zhang and Man Zhang collected and extracted the data. All authors read, provided feedback, and approved the final manuscript.

## CONFLICT OF INTEREST STATEMENT

The authors declare that the research was conducted in the absence of any commercial or financial relationships that could be construed as a potential conflict of interest.

## ETHICS STATEMENT

The authors declare that all methods were carried out in accordance with relevant guidelines and regulations. This study has been approved by the Ethics Committee of Children's Hospital of Chongqing Medical University (Ethical Review for Research No. 117; 2019). The informed consent of this study was obtained from all subjects' legal guardian(s).

## Data Availability

Data sharing not applicable to this article as no datasets were generated or analyzed during the current study.
